# Supplementary material for: Haplotype and diversity signatures of ultra-soft selective sweeps in HIV-1
Source: bioRxiv. 2026 Jul 22:2026.07.21.739925. Preprint. [Version 1] doi: 10.64898/2026.07.21.739925 (PMC13419785; doi:10.64898/2026.07.21.739925)
Supplement: 1 [file NIHPP2026.07.21.739925v1-supplement-1.pdf]

bioRxiv preprint doi: https://doi.org/10.64898/2026.07.21.739925; this version posted July 22, 2026. The copyright holder for this preprint (which was not certified by peer review) is the author/funder, who has granted bioRxiv a license to display the preprint in perpetuity. It is made available under a CC-BY 4.0 International license.

851 # Supplement

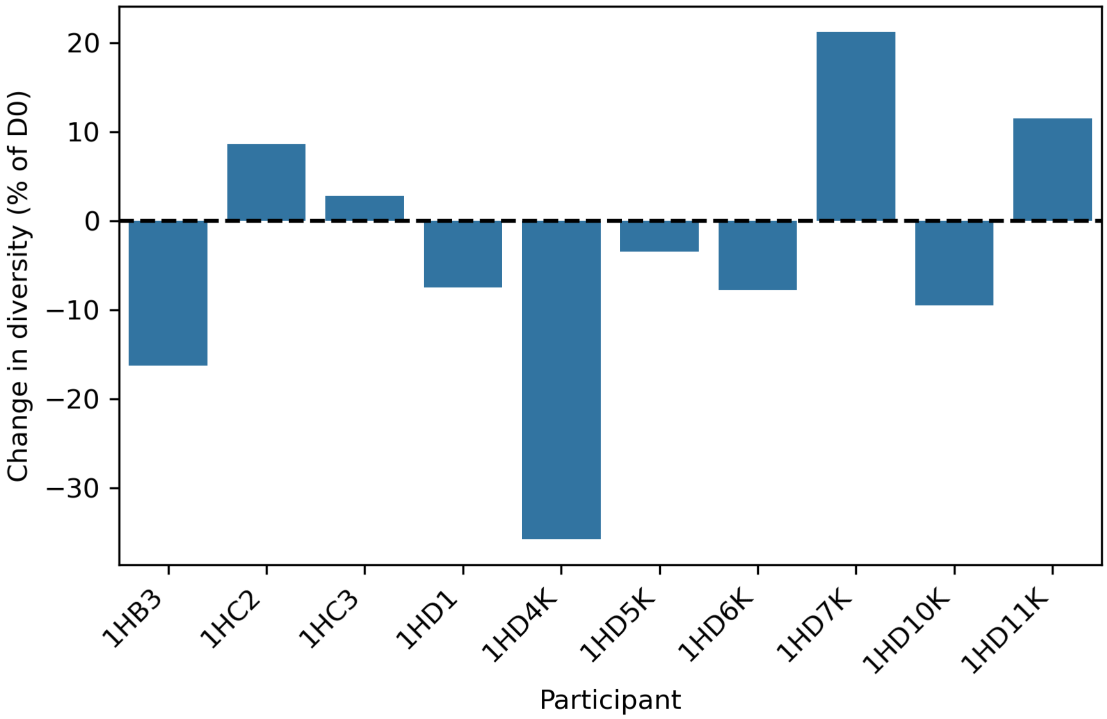

Figure S1: **Change in average pairwise Hamming distance across *env* following 10-1074 infusion.** Change in average pairwise Hamming distance across the full *env* gene between trial day and the first time point following viral load nadir is shown for each trial participant.

bioRxiv preprint doi: https://doi.org/10.64898/2026.07.21.739925; this version posted July 22, 2026. The copyright holder for this preprint (which was not certified by peer review) is the author/funder, who has granted bioRxiv a license to display the preprint in perpetuity. It is made available under a CC-BY 4.0 International license.

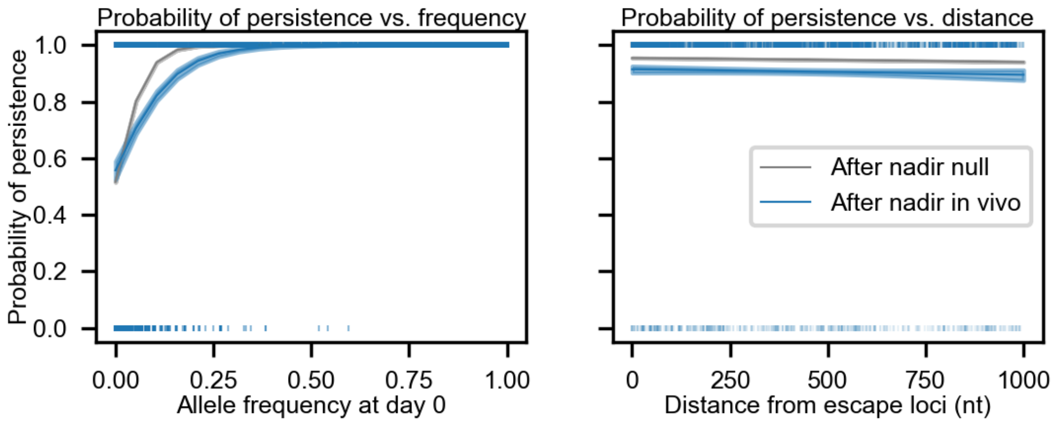

Figure S2: **Mutational probability of persistence following a sweep depends on pre-sweep frequency but not distance from the selected loci.** Tick marks at $y = 1$ or $y = 0$ indicate alleles segregating at day 0 and if they persisted or were lost, respectively, during the trial. The x-axis plots their day 0 allele frequency within a participant (left) or distance from the escape loci (right). Data from all participants is shown. Logistic regression fits to these data are plotted in blue. Null expectations of allelic loss based on the depth of sequencing at each time point are shown in gray (See Materials & Methods).

Table S1: **Parameters for gamma distributions fit to the simulated iiHH distributions that are shown in figure S7.**

Origins	Shape	Location	Scale
1	4.515	4.622	2.45
2	3.688	1.899	2.22
10	3.16	0.078	0.73
20	7.039	-0.97	0.292
30	31.794	-3.035	0.115
50	495.415	-9.479	0.02
100	500.594	-7.322	0.014

bioRxiv preprint doi: https://doi.org/10.64898/2026.07.21.739925; this version posted July 22, 2026. The copyright holder for this preprint (which was not certified by peer review) is the author/funder, who has granted bioRxiv a license to display the preprint in perpetuity. It is made available under a CC-BY 4.0 International license.

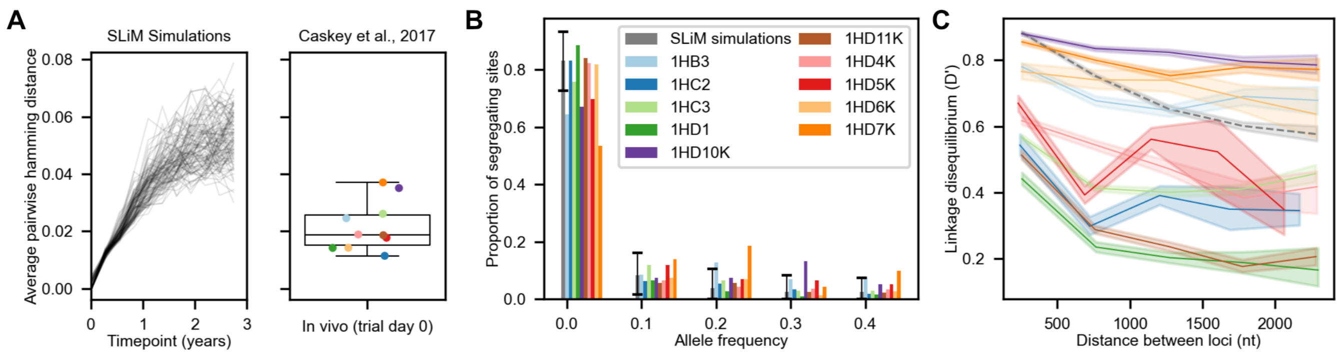

Figure S3: **Simulations match *in vivo* pre-treatment diversity, allele frequency spectra, and linkage decay. (A)** Left: Average pairwise Hamming distance over time in 100 simulated intra-host HIV populations. Right: Average pairwise Hamming distance in trial participants at trial day 0, before 10-1074 infusion [Caskey et al., 2017, Romero et al., 2026]. **(B)** Minor allele frequency spectrum for simulated intra-host populations (gray) and for 10-1074 trial participants at day 0 (colored). Simulated populations are sampled at generation 300 and error bars indicate 95% confidence intervals across 100 replicate simulations. Spectra are binned in increments of 0.1 . **(C)** Relationship between D' [Lewontin, 1988] and separating distance between pairs of segregating loci. The gray dashed line indicates the median linkage decay in 100 replicate simulations while the colored lines indicate the median linkage decay within each study participant. Shading represents 95% bootstrapped confidence intervals (1000 bootstrapped replicates).

bioRxiv preprint doi: https://doi.org/10.64898/2026.07.21.739925; this version posted July 22, 2026. The copyright holder for this preprint (which was not certified by peer review) is the author/funder, who has granted bioRxiv a license to display the preprint in perpetuity. It is made available under a CC-BY 4.0 International license.

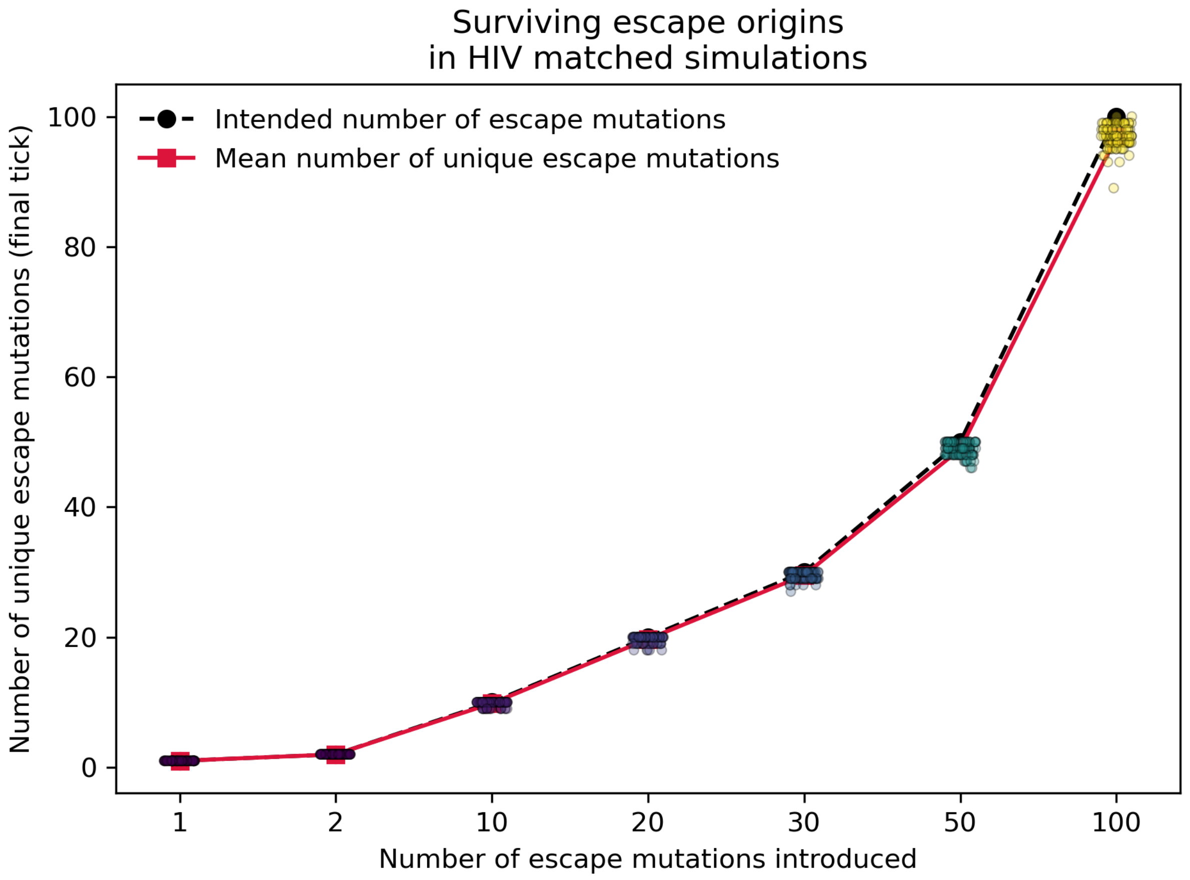

Figure S4: **Distribution of the number of surviving origins at sweep end in HIV matched simulations.** The number of surviving escape mutation origins 22 simulated generations ($\approx$ 8 weeks) after they were introduced. Simulations starting with 1, 2, 10, 20 30, 50, or 100 origins are included with 100 replicates shown for each scenario. Only simulations where the actual number or origins fell above a specified minimum number of origins and where the origins reached a cumulative frequency > 0.99 by the end of 22 generations were retained (See Materials & Methods).

bioRxiv preprint doi: https://doi.org/10.64898/2026.07.21.739925; this version posted July 22, 2026. The copyright holder for this preprint (which was not certified by peer review) is the author/funder, who has granted bioRxiv a license to display the preprint in perpetuity. It is made available under a CC-BY 4.0 International license.

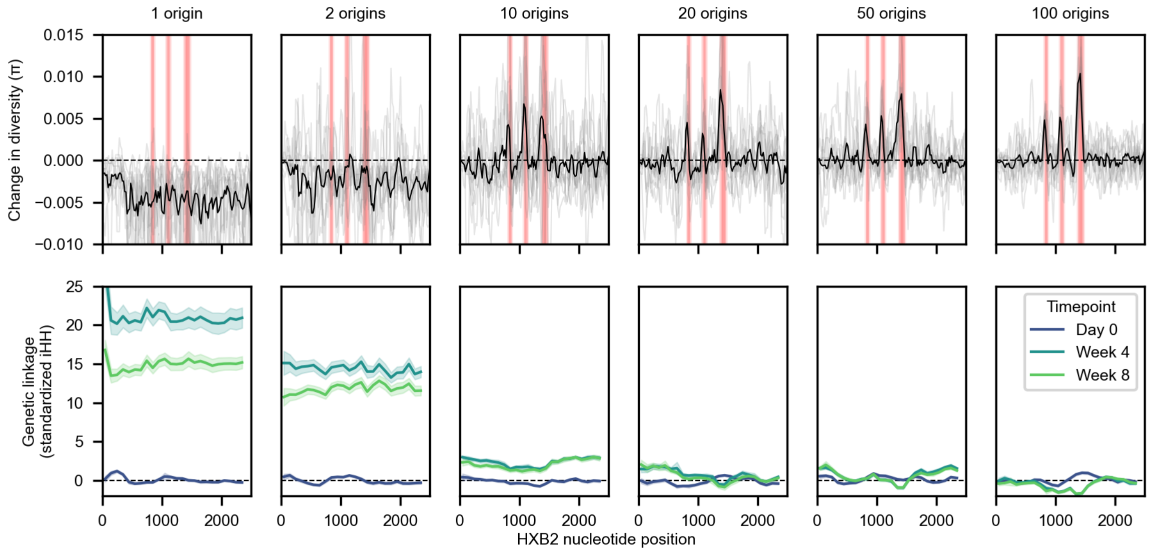

Figure S5: **Simulated sweeps of alleles at multiple escape loci.** Linkage and diversity changes are shown for sweeps of escape mutations spread across three different possible escape regions, indicated via red shading in the top panels. Sequences were limited to carrying a single beneficial escape mutation. **(Top)** Change in average pairwise Hamming distance between trial "day 0" and trial "week 4" (11 simulated generations later) for sweeps originating from 1 to 100 highly beneficial mutations introduced into randomly selected HIV genomes at a given escape locus within these three regions (see Materials & Methods). Individual simulation replicates are shown in light gray while the mean over 10 replicates is shown in black. Hamming distance is calculated in 60 nucleotide bins advanced by 20 nucleotides. The escape loci are shaded red. **(Bottom)** Standardized integrated haplotype homozygosity (iHH) plotted versus HXB2 nucleotide coordinate during simulated selective sweeps of different numbers of origins (Materials & Methods). Data are pooled across 10 replicates and plotted as a binned average (100 nucleotide positions per bin) with 95% confidence intervals on each bin.

bioRxiv preprint doi: https://doi.org/10.64898/2026.07.21.739925; this version posted July 22, 2026. The copyright holder for this preprint (which was not certified by peer review) is the author/funder, who has granted bioRxiv a license to display the preprint in perpetuity. It is made available under a CC-BY 4.0 International license.

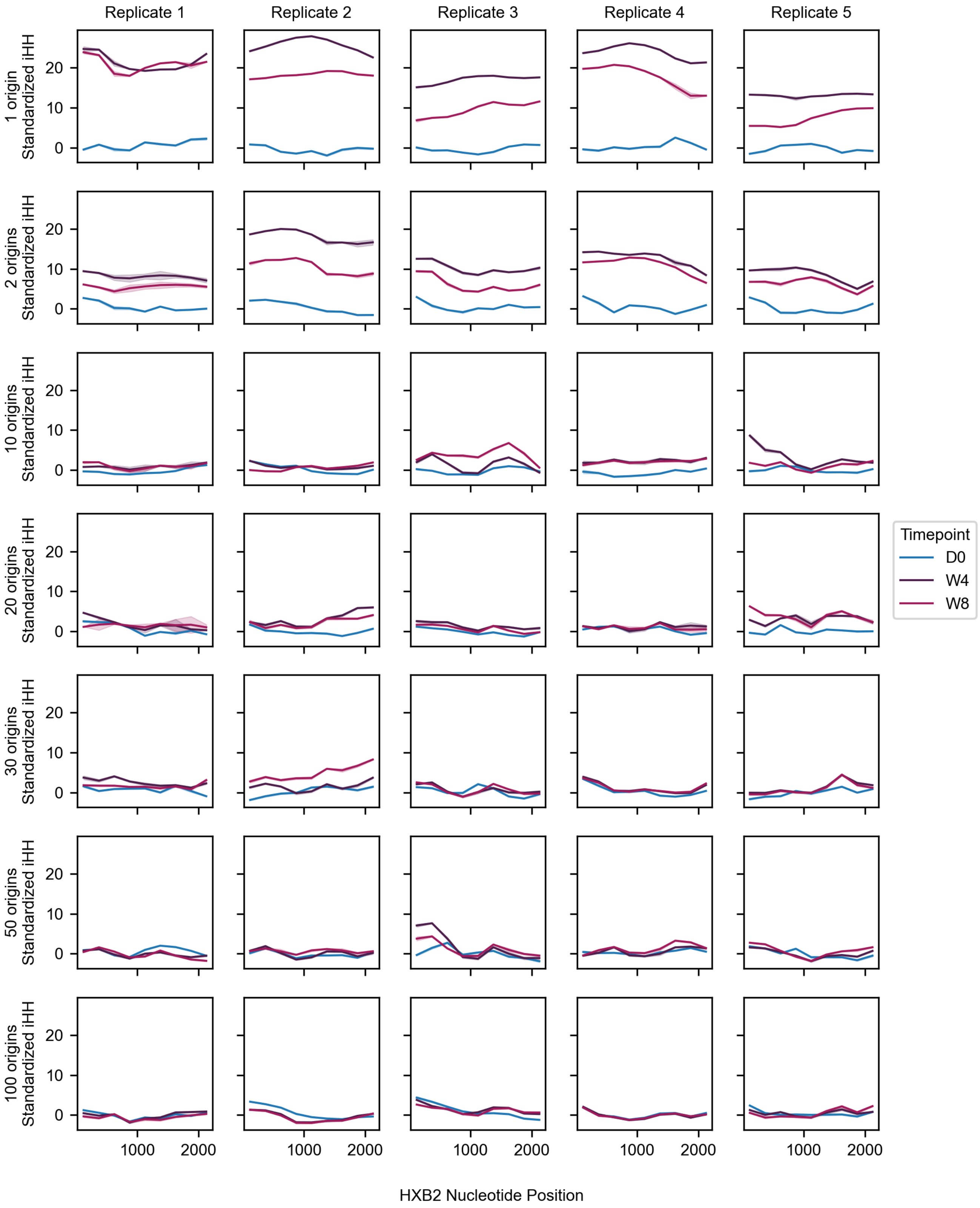

Figure S6: **Standardized iHH over time in single simulated replicates.** Standardized integrated haplotype homozygosity (iHH) is plotted versus HXB2 nucleotide coordinate during simulated selective sweeps (Materials & Methods). Each panel contains data for a single replicate of a simulation scenario with a given number of sweep origins. Five randomly selected examples are shown for each condition. Data are plotted as a binned average (100 nucleotide positions per bin) with 95% confidence intervals on each bin shown via shading.

bioRxiv preprint doi: https://doi.org/10.64898/2026.07.21.739925; this version posted July 22, 2026. The copyright holder for this preprint (which was not certified by peer review) is the author/funder, who has granted bioRxiv a license to display the preprint in perpetuity. It is made available under a CC-BY 4.0 International license.

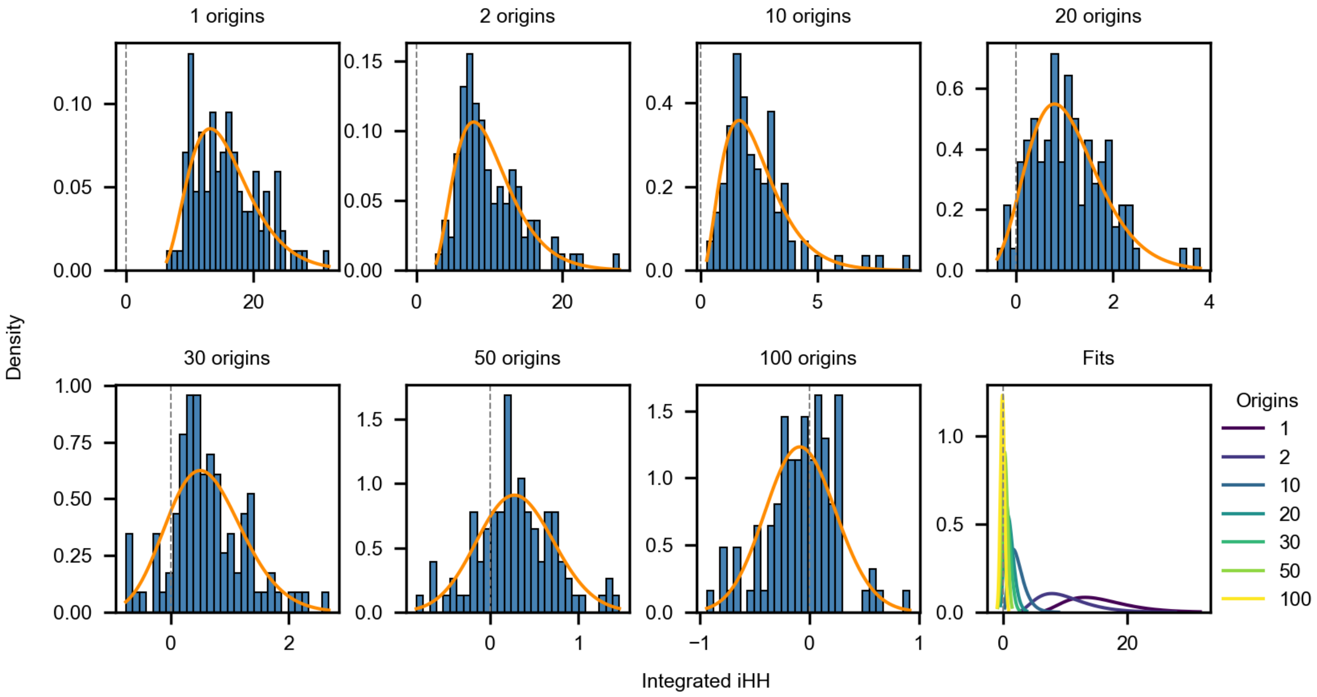

Figure S7: **Density plots of integrated iHH for simulated sweeps with set numbers of origins.** Selective sweeps simulated under HIV matched conditions are shown. Each density plot shows data from 100 replicate sweeps with a specific number of introduced origins. Orange curves indicate the gamma distribution fit to the corresponding simulated data (Table S1). The final panel shows the gamma distributions across all origin numbers for comparison.

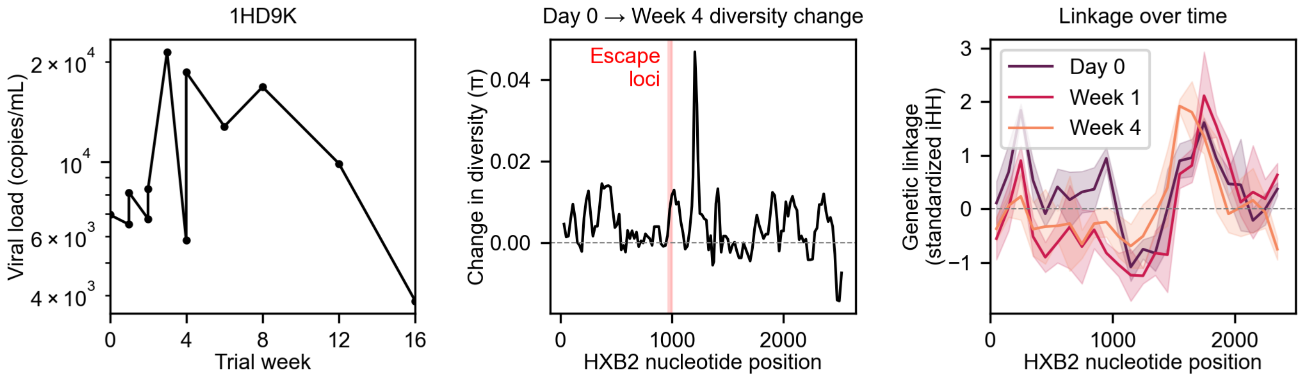

Figure S8: **Viral load, diversity, and iHH in non-responder 1HD9K during the 10-1074 trial.** **(A)** Viral load trajectory in 1HD9K throughout the 10-1074 trial. **(B)** Change in diversity in participant 1HD9K, measured via average pairwise Hamming distance between trial day 0 and trial week 4. **(C)** Standardized iHH is plotted versus HXB2 nucleotide coordinate for participant 1HD9K during the 10-1074 trial (Materials & Methods). Data are plotted as a binned average (100 nucleotide positions per bin) with 95% confidence intervals on each bin shown via shading.

bioRxiv preprint doi: https://doi.org/10.64898/2026.07.21.739925; this version posted July 22, 2026. The copyright holder for this preprint (which was not certified by peer review) is the author/funder, who has granted bioRxiv a license to display the preprint in perpetuity. It is made available under a CC-BY 4.0 International license.

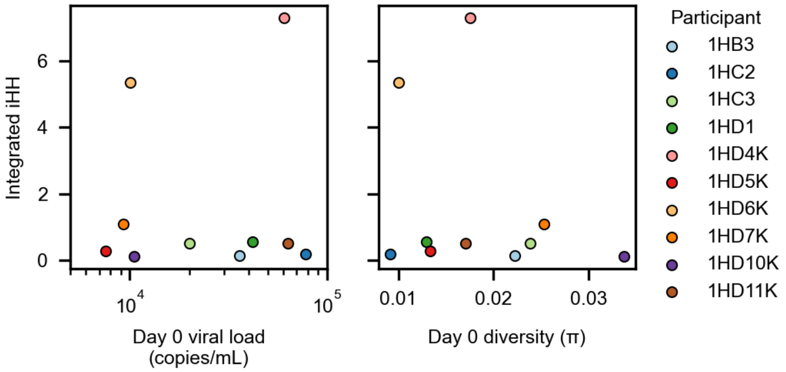

Figure S9: **Integrated iHH (iiHH) in 10-1074 trial participants compared to pre-infusion characteristics.** Integrated iHH for each trial participant is plotted in comparison to their viral load and intra-host HIV diversity pre-infusion, measured via *env* gene wide Hamming distance.

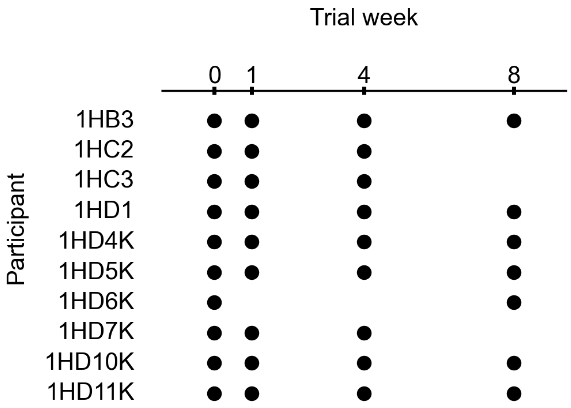

Figure S10: **Sampling time points of 10-1074 sequences analyzed in this investigation.** Dots denote all time points between trial day 0 and trial week 8 with available sequencing data.
